# Multispecific antibodies: Bioanalytics for early-stage screening and characterization of mispairing profiles

**DOI:** 10.1371/journal.pone.0336791

**Published:** 2025-11-20

**Authors:** Catarina Melo, Sofia B. Carvalho, Maria J. Sebastião, Ricardo A. Gomes, Pedro M. F. Sousa, Patrícia Gomes-Alves

**Affiliations:** 1 iBET, Instituto de Biologia Experimental e Tecnológica, Apartado, Oeiras, Portugal; 2 ITQB-NOVA, Instituto de Tecnologia Química e Biológica António Xavier, Universidade Nova de Lisboa, Oeiras, Portugal; University of Ferrara, ITALY

## Abstract

Multispecific antibodies (MsAbs) enable the targeting of different epitopes, representing a strategy with enhanced therapeutic potential. However, the intracellular assembly of MsAbs is complex and generates unwanted mispaired species, imposing a significant burden on downstream processing and analytical characterization, thus increasing the overall timeline and cost of bioprocess development. The establishment of analytical tools to aid in the characterization and understanding of MsAb mispairing profiles at early-stage screenings is mandatory. Here, we implemented mass spectrometry (MS) and high-performance liquid chromatography (HPLC) methods to assess mispairing levels using several Chinese Hamster Ovary (CHO) clones producing a MsAb. Results showed that both methods are suitable to be explored in early-stage screenings enabling the identification of higher quality MsAb producer clones. Importantly, not only protein A-purified but also clarified samples can be analysed by the methods established, streamlining the characterization process and reducing costs and analysis time. Moreover, we evaluated the impact of different mispairing levels on antibody functionality by biophysical tools. Nano-Differential Scanning Fluorometry (nDSF) was used to record thermal stability profiles and Surface Plasmon Resonance (SPR) to infer on the binary interactions established with three different antigens, revealing distinct profiles between groups with higher and lower mispairing levels. Our work allowed the development and implementation of a mispairing analytical toolbox, critical for early-stage screening and deeper characterization of these complex biopharmaceuticals.

## 1. Introduction

Monoclonal antibodies (mAbs) have been applied as a key therapeutic approach against cancer, autoimmune disorders, infectious diseases, and inflammatory conditions [1]. Over the last few decades, a new era of antibody-based therapeutics has emerged through the advent of multispecific antibodies (MsAbs). Compared to traditional monospecific antibodies, MsAbs possess multiple binding sites capable of recognizing two or more distinct epitopes, enabling simultaneous targeting of multiple antigens [2]. This multiple targeting capacity, higher specificity [3–5], and enhanced therapeutic potency, translates into an increased capability to cope with more complex diseases [6]. An increasing number of MsAbs are currently in preclinical and clinical development, with 399 ongoing studies at clinical development (*clinicaltrials.gov* accessed 18/08/2025, terms applied: “bispecific antibody”, “trispecific antibody” and/or “multi-specific antibody”; filters used, not yet recruiting, recruiting, active not recruiting, enrolling by invitation). So far, 17 MsAbs have been approved by the European Medicines Agency (EMA) and/or Food and Drug Administration (FDA) for clinical use [7–17] (*fda.gov*; *ema.europa.eu*).

MsAbs production requires the co-expression of distinct heavy (HC) and light (LC) chains. Consequently, incorrect chain pairing, generating undesired mispaired species often occurs [18]. Several strategies have been applied to prevent the formation of mispaired MsAb species through domain engineering [reviewed in [18–22]], such as CH3 domain engineering to prevent formation of HC homodimers (for example knob-into-hole strategy) and mutations in the Fragment Antigen-Binding (Fab) regions to prevent LC mispairing.

Despite the advances and approaches to ameliorate MsAb chain pairing in upstream manufacturing [19–21], mispairing remains a challenge. Mispaired species present distinct physico-chemical/structural features including distinct molecular masses, net charge, hydrophobicity, hydrodynamic diameter, thermal stability, propensity for aggregation, antigen binding affinity, and functional activity compared to the correctly paired antibodies [18,23]. These species are considered product-associated impurities that pose significant challenges to downstream processing (DSP), namely requiring extra purification steps to improve product quality [24]. This places a substantial burden on bioprocess development costs, as well as impacting overall yield [18]. Therefore, a set of analytical tools is essential for comprehensive product characterization at the mispairing level facilitating bioprocess optimization at this stage.

Screening for clones and process conditions that yield high quality (low misparing) antibody products is commonly performed only in the mid-late stages of development [25]. By characterizing the product at an earlier stage, we can filter for candidate clones preferentially producing low-mispaired antibodies, reducing costs and timelines.

Several analytical approaches can be employed to assess structural, functional, and biophysical mAbs' features [18]. Mass Spectrometry (MS) is a common tool used for detection, identification, and quantification of mispairing profiles in MsAbs. As an alternative routine quality control (QC) approach, HPLC [24] can be useful for separating and quantifying mispaired species. Biophysical methodologies are also employed for mAbs characterization, including structure, functionality, stability, and interactions assessment [26,27]. These properties can be evaluated through several techniques, including nano-Differential Scanning Fluorimetry (DSF), Surface Plasmon Resonance (SPR), and others [19,28].

**Fig 1:**
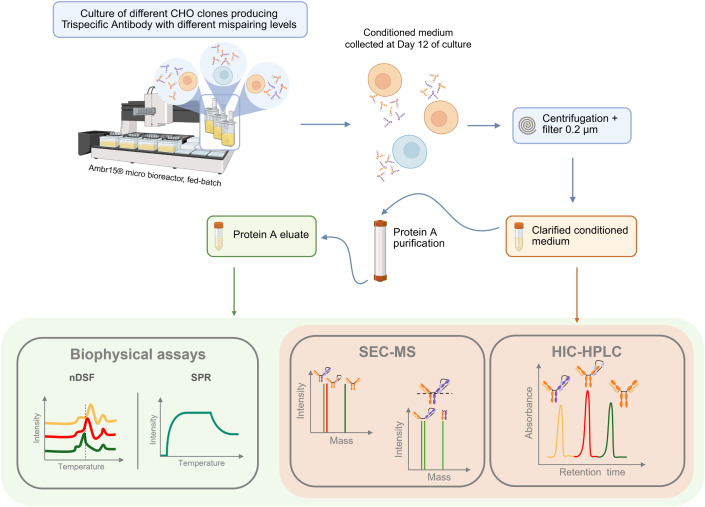
Mispairing characterization in distinct tsAb producer clones. Schematic representation of the experimental workflow used to evaluate mispairing profiles in CHO clones producing trispecific antibodies. Clones were cultured in Ambr15 micro bioreactors under fed-batch conditions. The conditioned medium collected on day 12 was clarified and filtered, followed by Protein A purification. The resulting eluates were subjected to bioanalytical characterization using biophysical assays (nDSF and SPR) and further analyzed by SEC-MS and HIC-HPLC.

Here, we report the development and implementation of a panel of bioanalytics aiming at characterizing several features of an anti-CD38/CD3 × CD28 trispecific antibody (tsAb) for early screening stages (Fig 1). We investigated the mispairing profiles and species of this tsAb produced by different CHO clones. Size Exclusion Chromatography MS (SEC-MS) was established for a detailed characterization of product quality. SEC-MS allows the identification and relative quantification of mispaired mAb species and other product impurities as free LC, HC and half-mAbs. The method can be applied to clarified samples, with no sample preparation. Hydrophobic Interaction Chromatography – High Performance Liquid Chromatography (HIC-HPLC) was applied as a higher-throughput QC method. Compared to SEC-MS, HIC analysis is faster with simpler data analysis and does not require costly equipment and highly specialized personnel. Therefore, it is more suitable for implementation in QC labs. Both SEC-MS and HIC-HPLC were used as complementary tools for the analysis of both non-purified bulk (clarified samples) and protein A purified samples. Additionally, advanced strategies involving biophysical tools for tsAb mispairing characterization were explored: a nDSF protocol was established to evaluate the impact of mispairing in tsAb thermal stability and SPR was used to assess functionality, through binding affinity and kinetics determination.

The methodological strategies set up in this work reinforce the analytical portfolio for MsAbs characterization, offering some QC-friendly and cost-effective approaches. The application of HIC-HPLC in clarified samples provides a quick and easy approach to distinguish between high mispairing and low mispairing profiles, streamlining the selection of the best clone candidates, i.e., producing higher quality tsAb.

## 2. Materials and methods

### 2.1. Cell culture and purification

#### 2.1.1. Trispecific antibody production using Ambr^®^ system.

A trispecific antibody (tsAb) was produced using a CHO cell line, by co-expression of four antibody subunits (LC1, LC2, HC1 and HC2) (provided by Sanofi). The producer cell clones were cultured for 12 days at a fed batch culture system process, in 15 mL microbioreactors from Ambr^®^ system (Sartorius). For each clone, duplicate or triplicate vessels were performed. The process parameters applied were the same for all replicates, which included initial cell density, pH control, feeding regime, temperature, and oxygen level. Viable cell density and viability were monitored using the Vi-Cell XR device (Beckman Coulter). Starting from culture day 5, conditioned medium was collected and centrifuged at 190 *g*, for 5 minutes, to analyze glucose, lactate, ammonia and IgG content through CedexBio analyzer (Roche). At day 12 (last day of culture), the entire cell culture supernatant was collected and centrifuged at 190 *g*, for 5 minutes at 4 °C. Day 12 centrifuged supernatant was clarified using a 0.22 µm PES filter unit (Millex-GP, SLGP033RS, MerckMillipore). Both clarified conditioned media and pellets were stored at −80 °C until further protein A purification and/or analysis.

#### 2.1.2. Protein A purification.

TsAb was purified using protein A affinity chromatography HiTrap MabSelect SuRe column (Cytiva). This step was performed using an AKTA Avant 25 system (Cytiva).

Protein A column was equilibrated with a PBS-based equilibration buffer, with a flow rate of 1.9 CV.min^-1^. Afterwards, clarified bulk was loaded, followed by a washing step with the same buffer until absorbance reached the baseline value. A second washing step was performed using a saline buffer to remove nonspecific binding interactions (e.g., ionic interactions). A new equilibration step was performed before elution. Elution was achieved using an Acetic Acid-based buffer (Elution buffer). Protein concentration of each fraction was determined by measuring the absorbance at 280 nm using the Lunatic equipment (Unchained Labs). Purified samples were frozen at −80 °C until further analysis.

### 2.2. Analysis of tsAbs by size-exclusion chromatography – mass spectrometry (SEC-MS)

All MS analyses were performed at UniMS – Mass Spectrometry Unit, iBET/ITQB-NOVA, Oeiras, Portugal. Samples were analyzed by LC-MS using the X500B Q-TOF (Sciex) coupled to the ExionLC (Sciex). Size-exclusion LC separation was performed using a UPLC BEH SEC column, 1.7 μm, 200 Å, 4.6 × 300 mm (Waters). The analyses were performed according to the method described elsewhere [23].

For clarified harvest samples (no protein A purification), 0.05% formic acid (FA) and 0.05% Trifluoroacetic acid (TFA) in 30:70 acetonitrile:water (LC-MS grade) in isocratic mode were used as the mobile phase. A flow rate of 0.1 mL.min^-1^ (from 0 to 27 min) and 0.4 mL.min^-1^ (from 27 to 37 min) was used. The valve was diverted to waste at 25.5 min. LC column was maintained at 25 °C and up to 50 µL injections were performed.

For the protein A-purified samples, a flow rate of 0.2 mL.min^-1^ for 20 min was used in isocratic mode with 0.1% formic acid (FA) in 30:70 acetonitrile:water (LC-MS grade). LC column was maintained at 25 °C and 5 µg injections were performed.

The X500B QTOF was used in positive ionization, intact protein mass, and large protein (>70 kDa) modes. TOF-MS analysis was done from 1000–6000 mz with 1 sec accumulation time. The parameters used were: 60 psi ion source gas 1, 40 psi ion source gas 2, 30 psi curtain gas, 5500 V ionization source voltage floating, 500 °C temperature of ion source (TEM), 80 times bins to sum, 180 V declustering potential, and 20 V collision energy. These parameters were used for both protein A purified and clarified samples.

Data was acquired with the Sciex OS (version 3.1.6, Sciex) and analyzed using the BioPharmaView (version 3.0, Sciex) software. In BioPharmaView software, the reference antibody (correct tsAb), possible mispaired species expected mass values and expected post-translational modifications were computed and used for automatic data analysis. The antibody species assignments considered a maximum mass tolerance of 10 Da and was manually confirmed. The processing parameters used are listed in S1 Table and the modifications considered are reported in S2 Table.

### 2.3. Hydrophobic Interaction Chromatography – High Performance Liquid Chromatography (HIC-HPLC)

Hydrophobic Interaction Chromatography – High Performance Liquid Chromatography (HIC-HPLC) was based on the method described elsewhere [26]. It was performed using the MAbPac HIC-10 column (5 μm, 4.6 × 250 mm; Thermo Fisher Scientific), in the 1260 Infinity II system (Agilent Technologies). The column temperature was maintained at 30 °C. Mobile phase A (MPA) was composed of 50 mM sodium phosphate, 1 M sodium sulfate, pH 6.5, and Mobile phase B (MPB) consisted of 50 mM sodium phosphate, pH 6.5.

The column was equilibrated with 100% MPA at a flow rate of 1 mL.min^-1^ until the UV signal stabilized. Clarified samples were diluted in MPA at a final concentration of 0.18 mg.mL^-1^ and protein A purified samples at 1 mg.mL^-1^. The volume injected for both sample types was 20 μL. HIC separation was performed using a linear gradient from 0% to 100% MPB in 30 min. After each sample run, a column washing step was performed using MPB (15 min), followed by an equilibration step using MPA (15 min). Protein signal was monitored at 280 nm. HIC-HPLC was operated using Chromeleon 7 (ThermoFisher) software, for instrument control and data handling. Main resolved peaks were manually integrated.

### 2.4. Nano differential scanning fluorimetry (nDSF)

nDSF analysis was performed on a Prometheus NT.48 instrument (NanoTemper Technologies GmbH). A human IgG1 (hIgG1), which does not interact with any of the selected antigens, was used as negative control. Protein A purified samples and hIgG1 were centrifuged for 30 min (4 °C, 17200 *g*) before analysis. The final reaction mixture contained 1 μg of each antibody sample at 100 µg.mL^-1^ diluted in the Elution buffer used in protein A purification step (section 2.2). High sensitivity capillaries (NanoTemper Technologies) were filled with 10 µl of each sample and placed on the sample holder. A temperature gradient of 1 °C.min^-1^ was applied from 20 °C to 95 °C and the intrinsic protein fluorescence at 330 and 350 nm was recorded. Data were analyzed using the value of the derived ratio 350/330 value. All samples were tested in triplicates.

### 2.5. Surface plasmon resonance (SPR)

SPR affinity and kinetic characterization of the binary interactions between tsAbs and the respective antigens was evaluated on a Biacore 4000 (Cytiva) at 25 °C.

Selected tsAb samples were diluted in HBS-P+ buffer (10 mM HEPES, pH 7.4, 150 mM NaCl, 0.05% Tween-20) to a final concentration of 500 ng.mL^-1^. Samples were directly injected on Protein A sensor chips (Cytiva), for 1–6 min, resulting in immobilization levels of 60–300 response units (RU). Three different antigens were separately injected over the immobilized tsAb surfaces at 10 different concentrations using a 2-fold dilution series during 220 s, followed by 660 s of running buffer flow (dissociation phase) at a flow rate of 30 µl/min. The selected antigens (human CD3 epsilon & CD3 delta (CD3ɛ&CD3δ) heterodimer (CDD-H52W1, Acrobyosytems), human CD28-His (CD8-H52HC, Acrobyosytems) and human CD38-His (CD8-h5224, Acrobyosytems) were injected at optimized concentrations, namely up to 1 nM for CD28, 100 nM for CD38 and 10 nM for CD3ɛ&CD3δ.

TsAb protein capture and binary interaction characterization with selected antigens were performed in presence of HBS-P+. Human IgG1, which does not interact with any of the selected antigens, was used as a negative control.

Biacore 4000 evaluation software was used to analyze and calculate all interaction kinetic parameters. All sensorgrams were processed by first subtracting the binding response recorded from the control surface (reference spot, without immobilized protein), followed by subtraction of the buffer blank injection from the reaction spot. All datasets were fitted to a simple 1:1 Langmuir interaction model to determine the kinetic rate constants, and the interaction affinity (K_D_) was calculated by dividing the dissociation rate (*k_d_*; s ⁻ ¹) and association rate (*k*_*a*_; M ⁻ ¹s ⁻ ¹)) constants. A Local Rmax was applied for those interactions which did not fully dissociate during buffer injection.

## 3. Results

### 3.1. SEC-MS for mispairing profiling and species identification

**Fig 2:**
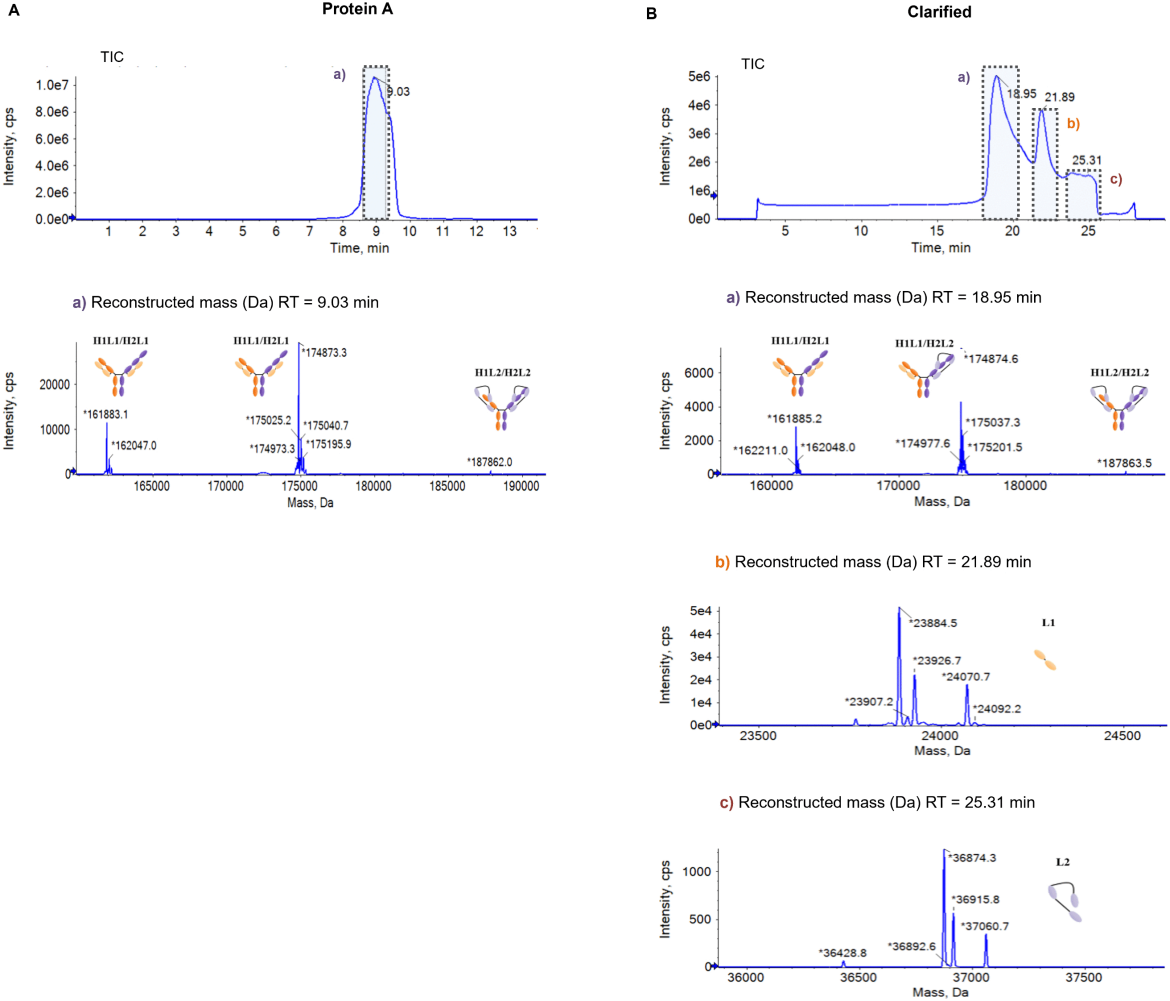
Total Ion Chromatogram (TIC) and respective reconstructed mass of the different peaks. (A) Protein A purified samples a) complete mAb species and **(B)** clarified samples a) complete mAb species, b) Light Chain 1 (L1) and c) Light chain 2 (L2).

**Fig 3:**
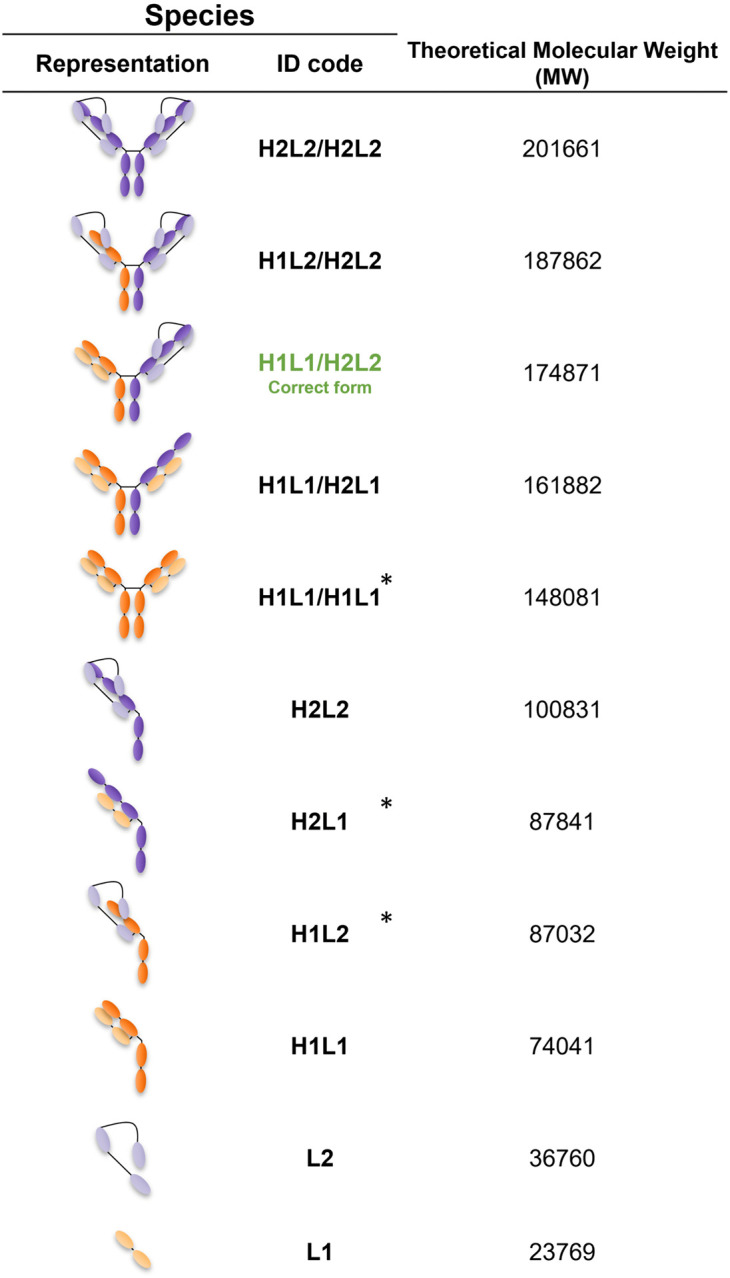
Correct and mispaired tsAb antibody species produced by CHO producers. H1L1/H2L2 – correct tsAb form (highlighted in green); H1L1/H2L1, H1L2/H2L2, H2L2/H2L2 – mispaired complete species; H2L2, H2L1, H1L1, H1L2 – half mispaired species. H = Heavy/L = Light chains. *Species not detected in SEC-MS analysis.

**Fig 4:**
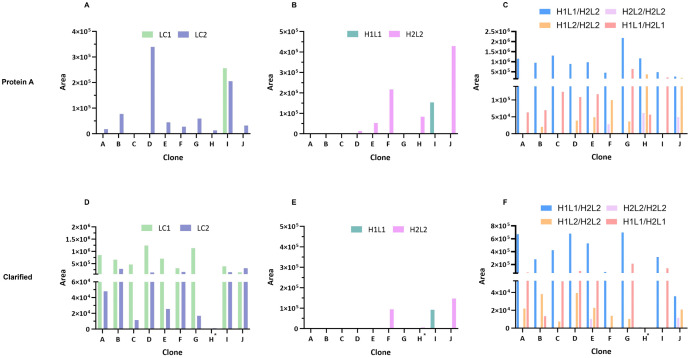
SEC-MS peak areas of Protein A purified and Clarified samples. Peak areas of **(A, B, C)** protein A purified samples and **(D, E, F)** clarified samples of all clones. Panels **A and D** depict the areas of free LCs detected (LC1 and LC2), panels **B and E** the half mAbs (H1L1 and H2L2) and panels **C and F** the complete mAbs (H1L1/H2L2 - correct form, H2L2/H2L2, H1L2/H2L2 and H1L1/H2L1). *No data available for clarified sample of clone H, as no MS signal was obtained.

We implemented a SEC-MS intact mass approach to characterize mispairing profiles, through the identification and quantification of the mispaired species contained in tsAb samples. A representative SEC-MS profile of Protein A purified sample and clarified sample are shown in Fig 2A and 2B, respectively. Free chains, half-mAbs and complete mAbs species were detected and are depicted in Fig 3. Protein A purified samples (Fig 4A–C) from cultures of 10 different CHO clones (A, B, C, D, E, F, G, H, I and J) producing the same tsAb were characterized. Additionally, the SEC-MS workflow allows the direct analysis of clarified samples (non-protein A purified), which streamlines the mispairing analysis process (Fig 4D–F).

**Fig 5:**
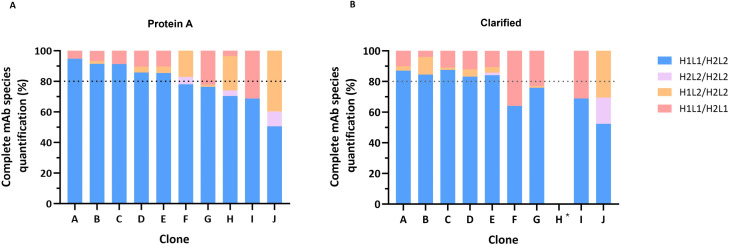
Type and distribution of tsAb complete species from distinct samples (A, B, C, D, F, G, H, I and J) analyzed by SEC-MS. Percentage of complete mAb species calculated for **(A)** Protein A purified and **(B)** clarified samples. H1L1/H2L2 corresponds to correct form (blue bars) and remaining mispaired species are depicted in other colors. Threshold line at 80% of complete mAb species. *No data available for clarified sample of clone H, as no MS signal was obtained.

These samples contained various species, including free light chain (LC) monomers, half-mAbs, and complete antibody. These species differ significantly in molecular weight, that carries important considerations for the analysis of MS data. Lower molecular weight species tend to ionize easily, presenting higher intensity MS signals, undervaluing larger species quantification (complete mAb) rather than accurately reflecting their abundance. To address this limitation, the individual peak areas for each protein species (free LCs, half mAbs and complete mAbs) identified in the SEC-MS analysis were reported. Relative quantitative analysis was only performed for mispairing species that have similar molecular weight (Fig 5).

For most clones, MS results indicate the presence of free LC monomers in both protein A purified and clarified samples (Fig 4A and 4D, respectively). LC1 is present only in clarified samples except for clone I, where LC1 is detected in protein A purified sample as well. However, LC2 is identified in both sample types (except in clone C purified sample). Additionally, half-mAb species are also observed in several clones, with higher peak areas for clones F, H, I and J (Fig 4B and 4E). H1L1 half-mAb is exclusively found in clone I. Regarding complete mAb species, we identified the correct antibody format (H1L1/H2L2) and other mispaired species (H1L1/H2L1, H1L2/H2L2, and H2L2/H2L2) (Fig 4C and 4F). For protein A purified samples of clones F, H and J, it is possible to observe higher peak areas corresponding to mispaired species H2L2/H2L2 and H1L2/H2L2.

Regarding the relative quantification of antibody mispairing, in protein A purified samples, higher levels of correct H1L1/H2L2 antibody were observed for clones A, B, C, D and E, with percentages above 80%. The remaining clones (F, G, H, I and J) presented percentages of H1L1/H2L2 below 80% (Fig 5A), with clone J presenting less than 50% of correct mAb form.

For clarified samples, the quantification profiles show a similar tendency when compared to protein A purified samples. Clones A, B, C, D and E showed higher levels of H1L1/H2L2 (above 80%) and F, G, I and J presented lower levels of the correct tsAb form, as observed for protein A purified samples.

Overall, for clones F, H, and J, protein A purified samples show higher levels of mispaired H2L2/H2L2, H1L2/H2L2 and H2L2 half-mAbs peak areas compared to the other clones (Fig 4B and 4C). In clarified samples of those clones, there is a lower area for the correct mAb form and higher H2L2 peak areas (Fig 4E and 4F). Clone I, on the other hand, has higher levels of H1L1 half-mAb and H1L1/H2L1. Considering only complete species, H1L2/H2L2 and H1L1/H2L1 were the prevalent mispaired forms observed (Fig 5). For protein A purified of clones F, H and J, H1L1/H2L1 was absent or observed at very low levels (Fig 5A). H2L2/H2L2 was mainly observed for clone J, for both protein A and clarified samples.

Based on the results shown in Fig 5A, the clones can be grouped into two profiles: lower mispairing, below 20% of mispairing (clones A, B, C, D, and E) and high mispairing, above 20% of mispairing (clones F, G, H, I, and J). Moreover, when considering all species identified by SEC-MS (free chains, half-mAbs and complete mAb), we observed that clones with higher half-mAb (Fig 4B and 4E) present higher levels of mAb mispairing (clones F, H, I, and J). However, no half-mAb species were identified for clone G.

### 3.2. HIC-HPLC as a high-throughput tool for mispairing profiling

Having established the SEC-MS method to accurately identify the different mAb species present in the different samples, we also evaluated an HIC-HPLC method, as a more suitable tool for high-throughput screenings.

**Fig 6:**
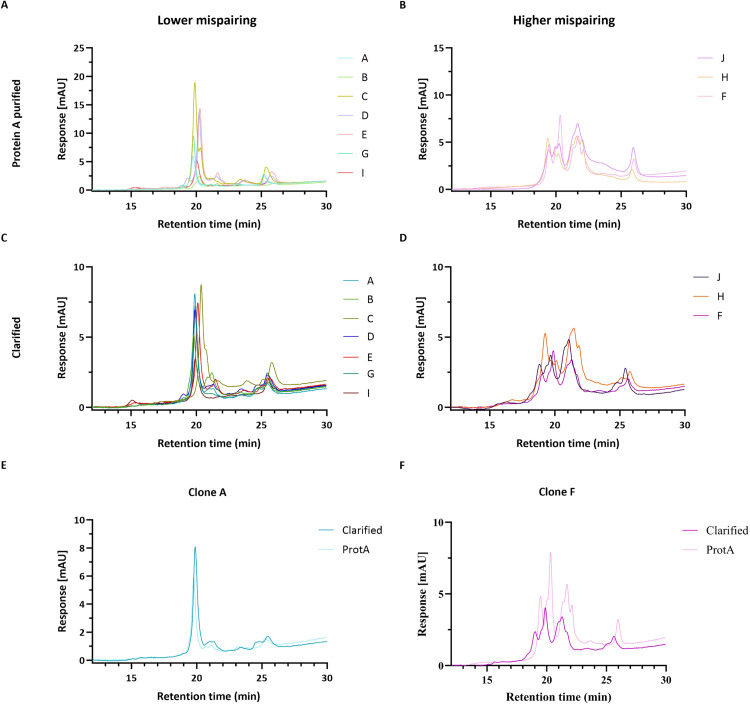
HIC-HPLC chromatograms of Clarified and Protein A purified samples for both mispairing profiles. Chromatogram overlay of **(A, B)** protein A purified samples and **(C, D)** clarified samples of clones for all clones. Chromatogram overlay of clarified and protein A purified samples for Clone A **(E)** and clone F (**F**), as examples.

Fig 6 panels A-D depict the chromatograms of protein A and clarified samples for all clones analyzed. Protein A purified samples were first used to evaluate the suitability of HIC-HPLC method to discriminate mispairing profiles. Several peaks were observed (Fig 6A and 6B), suggesting the presence of different tsAb species. It was possible to group samples in two distinct profiles: profile 1, including clones A, B, C, D, E, G and I, (Fig 6A); and profile 2, including clones F, H, J (Fig 6B).

We further optimized the HPLC protocol to analyze clarified samples, with no prior purification or sample preparation (Fig 6C and 6D). Chromatogram’s overlay of clarified and protein A purified samples shows remarkably similar profiles (Fig 6E and 6F, examples for clones A and F).

The combination of both MS and HPLC data suggests that HPLC profile 1 corresponds to samples with lower mispairing, while profile 2 corresponds to samples with higher mispairing (high content of half-mAb species).

### 3.3. Biophysical characterization insights into tsAb stability and functionality

**Fig 7:**
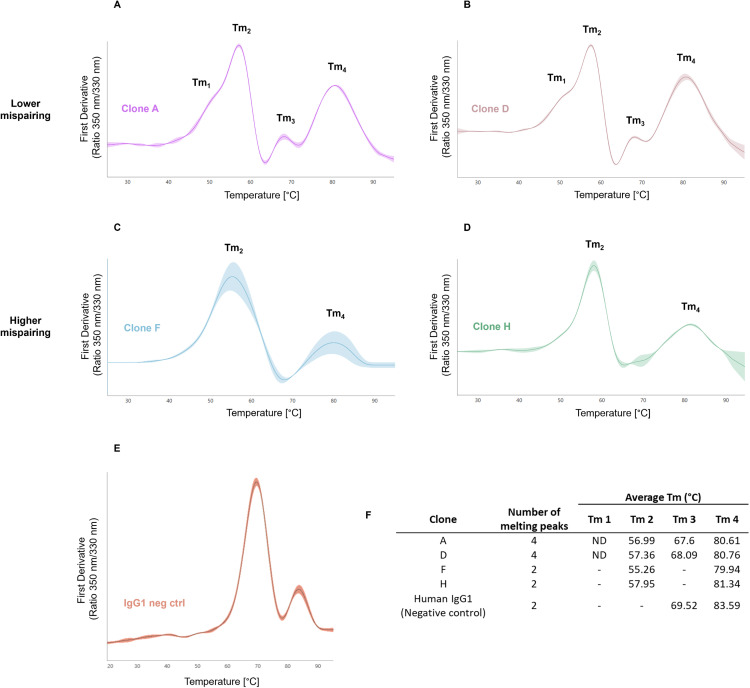
Characterization of tsAb thermal stability. nDSF profiles were obtained from four different clones within two different mispairing groups. **(A)** clone A and **(B)** clone D corresponding to lower mispairing and clone F **(C)** and clone H **(D)** to higher mispairing clone groups. **(E)** depicts the thermal stability profile of IgG1 negative control. The melting curves of each sample correspond to 350/330 nm first derivative and the curve shading reflects the standard deviation of the 3 replicates performed. (F) Average Tm values detected and number of melting peak transitions associated for all the clones evaluated, including human IgG1, used as negative control. ND – not determined.

Four out of the ten clones evaluated by SEC-MS and HIC-HPLC were selected for biophysical characterization by nDSF and SPR: two from the lower mispairing profile group (clones A and D) and two from the higher mispairing profile group (clones F and H). As these methodologies require high purity sample levels, only protein A purified samples were used. To evaluate mispairing impact on antibody conformation and its effect on protein stability [18], tsAb thermal stability by nDSF analysis was performed. Although the observed melting transitions (Fig 7) could not be assigned to any specific protein domains or sub-domains of the heterogeneous tsAb samples, two distinct profiles were clearly identified: 1) lower mispairing level group with four transitions (Tm_1_ to Tm_4_); 2) higher mispairing level group with two transitions (Tm_2_ and Tm_4_). A human IgG1 antibody, was assessed as a negative control (Fig 7E), presenting a distinct thermal stability profile when compared to the clones evaluated.

To evaluate the mispairing impact on antibody-antigen binary interaction affinity and kinetics, we characterized the selected samples by SPR. CD28, CD38 or CD3ε/CD3δ antigens were evaluated based on the literature reports related to this tsAb [27].

**Fig 8:**
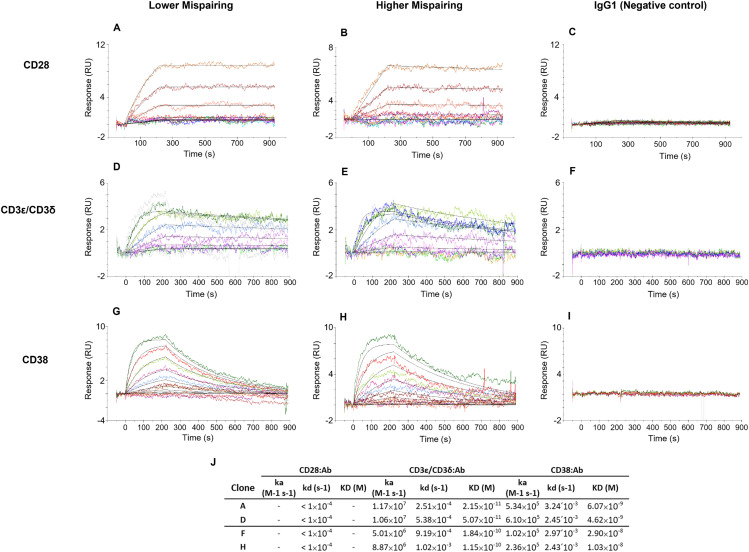
SPR kinetic characterization of the binary interaction between tsAb and each of the different antigens (CD28, CD3ε/CD3δ, CD38). Two different SPR sensorgram profiles were obtained for tsAb samples for all the clones from the different mispairing level groups. The figure depicts samples tested against: (A), (B) and (C) CD28, (D), (E) and (F) CD3ɛ/CD3δ, and (G), (H) and (I) CD38, each with ten different dilutions: up to 1 nM for CD28, 10 nM for CD3ɛ/CD3δ, and 100 nM for CD38. Two antibody clones are shown as examples: clone A, representing a lower mispairing clone and clone F representing a higher mispairing clone. **(C)**, **(F)** and **(I)** panels display the binding profile of igG1 negative control. **(J)** Binding kinetic parameters from CD28, CD3ε/CD3δ and CD38 binary interaction analysis. For CD28 antigen the dissociation rate was too slow to calculate the interaction affinity K_D_.

Binary interactions were successfully validated for the three antigens in all tested samples. For CD28:antibody (Ab) binding analyses, dissociation constant (*kd*) values obtained were too low, as the dissociation profile was too slow (*kd* < 1.0x10^-4^ s^-1^) preventing an accurate equilibrium dissociation constant (K_D_) calculation. These results suggest that CD28 has a very high affinity for all the evaluated samples (Fig 8A and 8B). Similar profiles were obtained for CD3ε/CD3δ (Fig 8D and 8E). However, the CD38:Ab binding analyses allowed us to detect minor differences between lower and higher mispairing groups, namely with respect to the calculated interaction affinities, which appear to be higher on the interaction with lower mispairing samples (Fig 8G, 8H, 8J). A human IgG1 antibody, which does not bind to any of the antigens examined in our study (CD28, CD3ε/CD3δ, CD38), was assessed as a negative control (Fig 8C, F, I).

## 4. Discussion

MsAbs bioprocess optimization requires strategies that improve product yield and quality while reducing costs. To cope with this challenge, mispairing characterization is critical, requiring a comprehensive set of analytical tools that aid in product characterization and impurities profiling.

Here, we established different bioanalytical tools to characterize mispairing in a tsAb product, focusing on: i) MS and HPLC for the identification and quantification of mispaired species profiles; ii) nDSF and SPR biophysical assays for assessing thermal stability and functionality.

MS should be applied for in-depth characterization of new MsAb molecules. This approach allows detailed quantification of the different complete antibody species and identification of low molecular weight species. After this initial step, HPLC can be used as a routine QC-friendly method for high-throughput clone’s screening, distinguishing lower vs higher mispairing signatures of different clones/conditions.

In this work, we established a SEC-MS approach for the analysis of protein A purified samples, enabling the identification of: free LCs monomers (LC1 and LC2), half-mAbs (H2L2 and H1L1) and complete mAb species ((H1L1/H2L2 – correct form, and H2L2/H2L2, H1L2/H2L2 and H1L1/H2L1 – mispaired species) (Fig 4, Fig 5). Considering complete mAb species, we can differentiate two groups based on the level of mispairing: those with lower mispairing (> 80% correct form) and those with higher mispairing (< 80% correct form). Additionally, SEC-MS can also be applied directly in clarified samples, allowing a faster characterization, but some challenges need to be taken into consideration. By skipping protein A purification, impurities and species lacking the Fragment Crystallizable (Fc) region are not removed. Although providing a more realistic overview of each sample composition, this results in the accumulation of high percentages of LC monomers. Generally, for traditional mAbs, expression vectors are constructed to favor excess expression of LC over HC, demonstrated to interfere with mAb pairing kinetics, improving antibody secretion and quality [28–30]. In MsAbs, the overabundance of LC may favor the formation of mispaired species, namely non-homologous LC-HC assembly [31].

Contrary to free LC2, which was present for most samples, LC1 was more prevalent in clarified compared to protein A purified samples (Fig 4A and 4D). During antibody purification step, protein A strongly binds to the HC Fc region (CH2-CH3 region), leading to the elution of species lacking Fc region or part of it. However, reported data shows that, although with low affinity, Fab region (that contains LC) also binds to protein A resin [32]. As free LC1 was mostly detected for clarified samples, it possibly binds with lower affinity to protein A compared to LC2. Additionally, half mAbs also bind weakly to protein A, as they only contain part of the Fc region [33]. This can also explain why we detect half mAbs for both clarified and protein A samples (Fig 4B and 4E).

The mispairing profiles of complete mAb are similar for protein A purified and clarified samples (Fig 4C and 4F). It is important to consider all the species being produced to determine if a clone has low or high mispairing profile. When considering all SEC-MS data, clones with higher half-mAb areas (F, H, I, and J) show higher levels of mispairing. Clone I has a unique profile due to the presence of a specific half-mAb species (H1L1). For this clone, we observed a higher amount of free LC1 after protein A purification, suggesting that this species is produced in larger quantities compared to other clones. This may also favor the formation of H1L1 half-mAb species.

After SEC-MS characterization, we further implemented an HIC-HPLC method as a high-throughput screening strategy. This approach allowed the successful identification of two mispairing profile groups, which aligned with the SEC-MS results, except for clone G and I. We observed that high mispairing samples containing a considerable amount of H2L2 (F, H and J) grouped in HPLC profile 2 (Figs 4, 6). Therefore, this species should have an important impact on HPLC profiles. Although clone G presents a SEC-MS profile that corresponds to the higher mispairing group, no H2L2 half-mAb species were identified for this sample. This may explain the resemblance of its chromatographic profile to HPLC profile 1 (associated with lower mispairing). Sample I profile is also different, maybe due to the specific half-mab species identified, H1L1, which is different from the half-mAb species found in other higher mispairing clones, explaining probably why its signature fits HPLC profile 1 (Fig 6). H1L1 is smaller than H2L2, with a different sequence, and its interaction with the HIC resin may vary due to different hydrophobicity.

The number of species detected by SEC-MS is distinct from the number of peaks detected in HIC-HPLC. This variation may be due to different method resolution and column chemistry, resulting in co-elution of some species or interference of other cell and medium components. Moreover, different species with distinct post-translational modifications also contribute to sample heterogeneity, interfering with the interaction with column matrix [34]. To accomplish an unbiased identification of HIC-HPLC peaks fraction collection followed by subsequent LC-MS analysis would be required.

HPLC method was further optimized for the analysis of clarified samples. This strategy maintains the capacity to discriminate between lower vs higher mispairing profiles, with a resolution similar to the protein A purified results (Fig 6). Notably, this method provides an alternative analysis option for early-stage screening of tsAb producer clones without the need of a purification step. The analysis of clarified samples was previously reported for bispecific mAbs, using ultra-high-performance liquid chromatography (RP-UHPLC) coupled with MS [35], but requiring protein A purification to perform RP-UHPLC as a stand-alone methodology. Our HIC-HPLC method enables the analysis of clarified samples without any previous purification steps, contributing to accelerating the timelines and decreasing associated cost..

A fast and cost-effective mispairing assessment is crucial in process development. Here we show that SEC-MS and HIC-HPLC can profile tsAb mispairing directly from clarified samples, bypassing the need for protein A purification, with results comparable to protein A-purified analyses.

TsAbs thermal stability and functionality characterization was performed by nDSF and SPR. Antibody stand-alone (without antigens) characterization by nDSF allowed the identification of lower vs higher mispairing profiles, that are also aligned with MS and HPLC data (Fig 7). This classification was based on the number of Tm transitions. The analysed tsAb’s FC region corresponds to an IgG4. Previous studies using an IgG4 monoclonal antibody showed that, at low pH values (pH = 5) three Tm value transitions occur during unfolding: Tm_1_ corresponding to the unfolding of the CH2 domain (less stable), Tm_2_ to the more stable CH3 domain, and Tm_3_ (the most stable) to the dissociation of the Fab region [36]. Considering that lower pH levels and different buffer formulations might impact observed Tm profiles, which were in elution buffer (pH = 3.2), this could explain the lower Tm observed compared to the reported data. Due to the heterogeneity and complexity of the tsAb samples, it is unclear which protein domain or sub-domains each Tm belongs to. Although most combinations exhibit a prevalent Ab type, this may not accurately represent the abundance of each species. For instance, the signal emitted for the less abundant Ab species can be higher if there is a higher content of tryptophan (which has aromatic heterocyclic rings that emit more fluorescence than other aromatic amino acids like tyrosine and phenylalanine) than the predominant Ab species detected [37,38]. Given the anticipated challenges related to melting curve changes in our heterogeneous samples, additional analysis/purification steps would be necessary to perform an accurate sample characterization, namely through chromatographic fractionation [18].

To assess tsAb functionality, binary interactions with CD28, CD3ε/CD3δ and CD38 antigens were evaluated by SPR (Fig 8). All samples showed binding, matching the previously observed distinct mispairing groups based on CD38 antigen interaction affinity: lower mispairing clones exhibited higher affinity, while higher mispairing clones displayed lower affinity. This discrepancy in interaction likely reflects the abundance of certain tsAb species, particularly H1L1-containing species, to which CD38 binds, and that are more common in lower mispairing clones (Fig 5 and Supplementary S1 Fig). While these results were shown to be specific for the selected antigens, the interaction assay here established can be applicable for other antibody types. Although SPR has been previously used for MsAb binding characterization [39], to our knowledge, this is the first report of SPR being used to qualitatively evaluate mispairing profiles through binary interactions.

## 5. Conclusions

**Fig 9:**
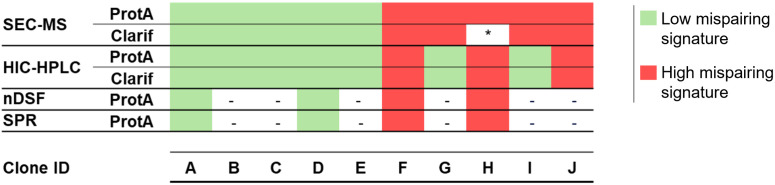
Summary of bioanalytical tools used for mispairing screening and characterization. ProtA – protein A purified samples; Clarif – Clarified samples. Color gradient corresponds to peak areas obtained in SEC-MS analysis: red gradient – LC’s, half-mabs and full mAbs mispaired species; green gradient – full mAb correct form. * No data available for clarified sample of clone H, as no MS signal was obtained.

In this project, we established distinct methodologies to assess mispairing profiles of a tsAb molecule, including SEC-MS, HIC-HPLC and biophysical assays, which can be adapted and used according to the expertise and equipment available in the laboratory. These approaches allowed the identification of distinct signatures between lower and higher mispairing profiles (Fig 9). This distinction appears to be predominantly influenced by the presence of half mabs and correct tsAb form. The implementation of these methods contributed also to a better understanding of the mispairing profile (mispaired species quantification and characterization). Remarkably, we were able to analyze clarified samples using a HIC-HPLC method compatible with high throughput, less time-consuming and lower costly analyses. To confirm the broader applicability of these methods for mispairing profiling, other molecules should be evaluated. The methodologies here implemented contribute to streamlining clone’s early screening process, and monitoring/control of product quality, thus reducing development & manufacturing costs and timelines.

## Supporting information

S1 Fig SPR kinetic characterization of the binary interaction between tsAb and each of the different antigens (CD28, CD3ε/CD3δ, CD38). Distinct SPR sensorgram profiles were obtained for tsAb samples for all the clones from the different mispairing level groups. The figure depicts two examples: (A), (B) and (C) correspond to clone D (clone with lower mispairing levels) and (D), (E) and (F) correspond to clone H (clone with higher mispairing levels). Each sample/antigen contains ten different dilutions: up to 1 nM for CD28, 100 nM for CD38 and 10 nM for CD3ɛ&CD3δ. (G) Binding kinetic parameters from CD28, CD3ε/CD3δ and CD38 binary interaction analysis. For CD28 antigen the dissociation rate was too slow to calculate the kD.
(TIF)

S1 Table Processing parameters used in SEC-MS. (PDF)

S2 Table Modifications analyzed in SEC-MS analysis. NeuAc – N-acetylneuraminic acid; * - No defined position in the sequence; n/a – not applicable.
(PDF)

S3 Table Retention time (minutes) and relative percentage (%) of each eluted peak detected in HIC-HPLC of protein A and clarified samples. (PDF)
