# Supplementary material for: Multispecific antibodies: Bioanalytics for early-stage screening and characterization of mispairing profiles
Source: PLoS One. 2025 Nov 20;20(11):e0336791. doi: 10.1371/journal.pone.0336791 (PMC12633938; doi:10.1371/journal.pone.0336791)
Supplement: S1 Table — (PDF) [file pone.0336791.s002.pdf]

Processing Settings

Matching Tolerance	10.00 Da
Start m/z	1000.00
Stop m/z	6000.00
Start Mass	153989.50 Da
Stop Mass	207639.25 Da
RT Range Processing	Time Selection
Start Time (min)	7.00
Stop Time (min)	12.00
Perform LC Peak Detection	On

Chromatographic Data Processing

Peak Threshold	≥ 1.00%
Gaussian Smoothing	1 points
Number of TOFMS Spectra to Combine	± 12 scans

Reconstruction Processing

Iterations	20
Signal to Noise Threshold	≥ 20.00
Resolution	2500
Gaussian Smoothing	0.00

Protein Results

Relative Result Threshold	≥ 1.00%
Maximum Number of Combined Modifications per Protein	20
