# Supplementary material for: Multispecific antibodies: Bioanalytics for early-stage screening and characterization of mispairing profiles
Source: PLoS One. 2025 Nov 20;20(11):e0336791. doi: 10.1371/journal.pone.0336791 (PMC12633938; doi:10.1371/journal.pone.0336791)
Supplement: S2 Table — NeuAc – N-acetylneuraminic acid; * - No defined position in the sequence; n/a – not applicable. (PDF) [file pone.0336791.s003.pdf]

Type	Name	Position	Maximum Mods per Chain	Modified AA	Applies to	Workflow usage	Mass Shift (Da)
Internal	G0F	421	-	N	N	Intact Protein	1444.53
N-terminal	Gln -> pyro-Glu	-	-	Q	Q	Intact Protein	-17.03
Internal	G1F	421	-	N	N	Intact Protein	1606.59
Internal	G2F	421	-	N	N	Intact Protein	1768.64
Internal	G0F	297	-	N	N	Intact Protein	1444.53
Internal	G2F	297	-	N	N	Intact Protein	1768.64
Internal	G1F	297	-	N	N	Intact Protein	1606.59
Internal	NeuAc	*	1	n/a	STN	Intact Protein	291.10
