# Supplementary material for: Multispecific antibodies: Bioanalytics for early-stage screening and characterization of mispairing profiles
Source: PLoS One. 2025 Nov 20;20(11):e0336791. doi: 10.1371/journal.pone.0336791 (PMC12633938; doi:10.1371/journal.pone.0336791)
Supplement: S3 Table — (PDF) [file pone.0336791.s004.pdf]

		Peaks (ordered by retention time)																					
		1		2		3		4		5		6		7		8		9		10		11	
**Sample**	**Sample type**	**RT (min)**	**%**	**RT (min)**	**%**	**RT (min)**	**%**	**RT (min)**	**%**	**RT (min)**	**%**	**RT (min)**	**%**	**RT (min)**	**%**	**RT (min)**	**%**	**RT (min)**	**%**	**RT (min)**	**%**	**RT (min)**	**%**
**A**	**Protein A**	19.76	70.84	21.14	10.70	24.39	5.06	25.26	13.40	-	-	-	-	-	-	-	-	-	-	-	-	-	-
**B**		18.96	3.65	20.24	57.65	21.17	8.53	21.64	17.11	23.67	1.98	25.96	11.08	-	-	-	-	-	-	-	-	-	-
**C**		19.89	49.50	20,31	18.86	21.21	8.24	22.28	2.49	23.39	7.66	25.37	13.27	-	-	-	-	-	-	-	-	-	-
**D**		19.35	7.81	20.21	54.82	21.13	5.13	21.61	8.25	21.98	6.40	23.66	7.83	25.69	9.75	-	-	-	-	-	-	-	-
**E**		15.48	1.42	18.28	0.59	20.28	56.88	20.96	0.21	21.64	16.44	22.80	2.05	23.78	8.44	25.78	13.97	-					
**F**		19.49	15.07	19.98	7.83	20.33	21.73	21.39	11.53	21.71	15.05	22.11	13.84	23.69	4.79	24.61	2.07	25.98	8.07	-	-	-	-
**G**		18.91	2.83	19.77	67.82	21.16	4.82	21.60	2.29	23.27	4.27	25.20	17.97	-	-	-	-	-	-	-	-	-	-
**H**		19.38	17.57	19.87	7.47	20.20	10.31	21.18	12.87	21.60	13.58	21.98	20.59	23.36	3.98	23.80	5.90	25.83	7.73	-	-	-	-
**I**		15.30	5.95	16.38	2.24	20.06	82.58	25.49	9.23	-	-	-	-	-	-	-	-	-	-	-	-	-	-
**J**		19.44	12.68	19.96	6.93	20.24	10.02	21.26	14.07	21.66	13.97	22.10	17.31	23.51	14.59	25.94	10.42	-	-	-	-	-	-
**A**	**Clarified**	19.88	66.14	21.31	14.60	23.45	2.00	24.88	6.00	25.44	11.26	-	-	-	-	-	-	-	-	-	-	-	-
**B**		20.37	37.22	20.67	14.36	21.72	14.76	23.98	12.76	25.32	4.78	25.79	16.11	-	-	-	-	-	-	-	-	-	-
**C**		19.80	52.50	20.87	12.52	21.19	18.95	23.22	1.98	25.09	5.06	25.55	8.98	-	-	-	-	-	-	-	-	-	-
**D**		19.03	3.93	19.90	50.85	21.07	6.41	21.28	7.58	21.68	3.04	22.46	3.01	23.54	6.51	25.14	6.43	25.46	12.23	-	-	-	-
**E**		15.82	1.38	17.76	0.59	20.11	57.72	21.46	20.95	23.72	4.65	25.21	3.95	25.65	10.75	-	-	-	-	-	-	-	-
**F**		19.03	13.78	19.63	6.89	19.89	21.04	21.07	13.55	21.30	15.40	21.60	10.32	23.54	6.68	24.66	1.81	25.26	3.23	25.62	7.28	-	-
**G**		19.30	3.47	19.93	58.67	21.29	7.90	21.85	2.49	23.65	4.80	24.96	5.22	25.46	17.46	-	-	-	-	-	-	-	-
**H**		18.58	3.51	19.26	18.42	19.76	6.00	20.12	9.64	21.25	14.19	21.46	15.28	21.87	12.78	22.62	7.51	24.61	2.80	25.19	4.57	25.77	5.29
**I**		15.13	5.85	19.92	62.51	22.27	4.30	23.53	3.47	25.46	23.87	-	-	-	-	-		-	-	-	-	-	-
**J**		18.83	15.10	19.32	6.60	19.68	15.05	20.72	16.88	21.07	14.58	21.54	8.93	22.25	5.79	24.88	8.12	25.42	8.95	-	-	-	-
